# A Survey of Health-Related Activities on Second Life

**DOI:** 10.2196/jmir.1192

**Published:** 2009-05-22

**Authors:** Leslie Beard, Kumanan Wilson, Dante Morra, Jennifer Keelan

**Affiliations:** ^3^Dalla Lana School of Public HealthUniversity of TorontoTorontoONCanada; ^2^Department of MedicineOttawa Health Research InstituteUniversity of OttawaOttawaONCanada; ^1^Centre for Innovation in Complex CareUniversity Hospital NetworkTorontoONCanada

**Keywords:** User-computer interface, virtual systems, virtual worlds, Second Life, health education, social and behavioral research, behavioral research, health behavior, health behavior change

## Abstract

**Background:**

Increasingly, governments, health care agencies, companies, and private groups have chosen Second Life as part of their Web 2.0 communication strategies. Second Life offers unique design features for disseminating health information, training health professionals, and enabling patient education for both academic and commercial health behavior research.

**Objectives:**

This study aimed to survey and categorize the range of health-related activities on Second Life; to examine the design attributes of the most innovative and popular sites; and to assess the potential utility of Second Life for the dissemination of health information and for health behavior change.

**Methods:**

We used three separate search strategies to identify health-related sites on Second Life. The first used the application’s search engine, entering both generic and select illness-specific keywords, to seek out sites. The second identified sites through a comprehensive review of print, blog, and media sources discussing health activities on Second Life. We then visited each site and used a snowball method to identify other health sites until we reached saturation (no new health sites were identified). The content, user experience, and chief purpose of each site were tabulated as well as basic site information, including user traffic data and site size.

**Results:**

We found a wide range of health-related activities on Second Life, and a diverse group of users, including organizations, groups, and individuals. For many users, Second Life activities are a part of their Web 2.0 communication strategy. The most common type of health-related site in our sample (n = 68) were those whose principle aim was patient education or to increase awareness about health issues. The second most common type of site were support sites, followed by training sites, and marketing sites. Finally, a few sites were purpose-built to conduct research in SL or to recruit participants for real-life research.

**Conclusions:**

Studies show that behaviors from virtual worlds can translate to the real world. Our survey suggests that users are engaged in a range of health-related activities in Second Life which are potentially impacting real-life behaviors. Further research evaluating the impact of health-related activities on Second Life is warranted.

## Introduction

Second Life, created by San Francisco based company Linden Labs in 2003, is an online virtual reality world where users, called residents, create their own virtual selves, called avatars, and interact within a simulated 3-D environment, literally living a virtual “Second Life”. Users can register for a free basic account online at Second Life’s website [1] and download the free software to run the program. Users can search for places to visit, people to meet, and groups to join, and they can even create their own objects and spaces using software provided by Second Life. The content in Second Life is all user-generated.

There are over 15 million registered users within the virtual world, and over 1 million active users. Statistics from December 18, 2008 indicate that 1,437,910 residents had logged in to Second Life within the previous sixty days [2]. The average age of a Second Life user is 32, and there are slightly more males than females using the platform [3]. Second Life has its own currency called the Linden dollar which is traded on the LindeX. Linden dollars can be traded for real world currency, based on fluctuating market values. At the time this article was written, the exchange rate was approximately LD $263 to US $1 [4]. Similar to the real world, there is a spatial geography to Second Life where users can freely interact. There are over 19,000 islands in Second Life [3] which users navigate by making their avatars walk, run, fly, or teleport directly from place to place. Users can also wander through the world; however, there are private spaces with restricted access.

The communication features in Second Life simulate real world communication. For example, sounds become louder as the avatar moves closer to the source. Avatars can publicly or privately chat with each other either through voice or text tools. When using text chat, avatars automatically become animated and type in the air as their user enters a message. Instant messaging and note cards — text files that users receive when entering certain spaces — are other ways that people can communicate with each other both online and offline. Avatars also come with a variety of common human gestures that can be activated by the user, allowing them to communicate with virtual body language

Second Life encourages anonymity and interactivity, and it can be accessed from any location with a high-speed Internet connection. This makes it a potentially effective tool for disseminating user-generated health information on a site where users can access, learn about, and discuss various health topics. Many different health care agencies, organizations, companies, and private groups have chosen Second Life as one of their Web 2.0 communication strategies.

There are few research articles describing or surveying the range of health-related activities on Second Life [5,6,7,8]. A notable exception is Kamel Boulos et al’s (2007) overview of several health sites, “Second Life: an overview of the potential of 3-D virtual worlds in medical and health education” [7]. Kamel Boulous has also compiled a list of Second Life health resources on a website Health Cybermap [9]. Hansen (2008) described the potential of 3-D health care learning environments on Second Life in her review of existing literature [6]. Other Web 2.0 sites describe a range of health resources in Second Life, such as Meskó’s blog, “Top 10 Medical Sites in Second Life” [10] and websites that provide direct links (SLurls — Second Life URLs) to sites in the program. SL Healthy is an online wiki where people can submit information about their health site in Second Life [11]. However, to our knowledge, this is the first comprehensive survey of health activities on Second Life. This article not only catalogues the kinds of health-related activities within Second Life, but also summarizes and compares the various strategies used to communicate health information in a virtual world setting.

## Methods

To explore Second Life, we opened a free basic account and created an avatar, named Ellebee Helendale. We registered for a username and password, and then downloaded and installed the free software. The Second Life software contains an application-specific search engine; however, trial searches using a selection of generic and illness-specific keywords were disappointing (Table 1), yielding few relevant results. We focused on physical sites (called “Places” in the Second Life search engine), and identified several health sites. In addition to this search strategy, we identified several key Second Life locations through a comprehensive review of print, blog, and media sources discussing health activities on Second Life (using PubMed, Scholars Portal, Google, and Google Scholar). We then used a snowball method to identify other health sites until we reached saturation (no new health sites were identified). The search methods used to identify each site are coded in Table 2, Table 3, Table 4, Table 5, and Table 6 as follows: SLSRCH = Second Life Search Engine, CLR = Comprehensive Literature Review, SB = Snowball. We then visited sites with relevant descriptions and catalogued experiences from those with the most pertinent content. The first point of identification for each site is listed in bold in Table 2, Table 3, Table 4, Table 5, and Table 6. We described and analyzed our experiences and developed categories to group sites based on their primary purpose, and the primary purpose is listed in bold (Tables 2 - 6; Multimedia Appendix 1).

**Table 1:** Search results using a variety of test keywords in the Second Life search engine

Keywords	Relevant Hits (13 relevant of 68 total returns)
health^a^, hospital, American Cancer Society, Autism^a^, Autistic^a^, CDC Island^a^, vaccine^a^, vaccination, immunization, depression^a^, sexual health^a^, AIDs, HIV	American Cancer Society, Autism Parents’ Connection – SOS; Biomedicine Research Organization; Coordinated School Health for Teachers; Healthinfo Island; UIS/ CSU/ Basuah Welcome; University of Wisconsin-Milwaukee, Health Science, Virtual Cancer Institute; The Walk of Life Natural Health Education Center; CDC Island; Iowa Wellness and Spinal Tuning Center; NewWays – Counselling & Support; Palomar West Hospital; SL-Labs Psychology at University of Derby.

^a^Indicates a hit with this keyword.

We tabulated all sites that either disseminated health information or provided a health experience to users with some English content. Excluded were sites that did not directly disseminate health information or provide a health experience to users; for example, sites that served solely as memorials for victims of specific diseases but contained no information on the disease itself. We recorded how the site was found, including those found through a snowball (SB), through the Second Life search engine (SLSRCH), or through comprehensive literature review (CLR). Our searches often identified specially formatted hyperlinks, or SLurls, or the precise keywords that, once inputted into the Second Life search engine, provided a direct link to the Second Life site. We also summarized the features of the identified sites, traffic information, region size, and region name. Traffic is defined as the number of minutes a unique avatar spends on an area of land [12] and is ultimately a rough measure of site popularity. Measuring site usage, or popularity, by tracking user traffic is inexact; however, other measures such as the user “count statistics” are even more unreliable due to user gaming and manipulation (eg, installing dummy avatars or “bots” permanently in the site, or paying users to keep their avatar at the site, a practice also known as “camping”). To compare the traffic between different health sites, we recorded the traffic for each site on the day of December 18, 2008 in a randomized order. This measure is only a snapshot of the popularity of each site when we visited it and does not reflect fluctuations in traffic based on Second Life events and meetings nor different times of the day.

The region information recorded includes the region name (the host of the site) and the size. Region size is measured in square meters and can be used to compare the geographical size of each site. Multiple unique sites can exist in a single region [13]. The region size can also fluctuate if more real estate is purchased. As with traffic, we recorded the region size and name for each site on the day of December 18, 2008.

We found five distinct types of health-related activities on Second Life and classified them as follows:

### Education & Awareness

These sites focused primarily on offering information about various health issues, redirecting users to other websites and real-life information centers. Many of these sites also included discussion groups, lectures, classrooms, and events for communicating information about specific topics.

### Support

Sites in this category often offered one-on-one discussion with real-life doctors, therapists, nurses, librarians, and other health care professionals. Some sites also facilitate peer support groups, both moderated and not, with specific topics, group membership, events, and meeting places.

### Training

Training sites focus on educating people in the health care industry. Some sites are specific to the type of training they provide, and offer classrooms, discussions, lectures, simulations of health experiences, and patient interactions. Training sites that are linked to schools sometimes offer real-life academic credit for training completed within the Second Life site.

### Marketing & Promotion of Health Services

These sites exist primarily to promote new or future health services, organizations, fundraising efforts, and real-life health care initiatives. Some sites offer users an experiential simulation of an organization’s future plans for health care, while others recruit real-life volunteers for fund raising projects.

### Research

These sites are actively engaged in recruiting participants and conducting health research in both Second Life and real world settings.

While these categories reflect what we found to be the primary motivations for these Second Life sites, many sites fall into multiple categories, offering multiple experiences.

## Results

We found 68 relevant health sites that we included in our sample. In our experience, the application’s search engine failed to return many relevant sites with generic keywords (13 relevant sites/68 total sites sampled). The most successful way of identifying sites was through a comprehensive literature review. This was the first point of identification for 47 relevant sites out of 68 total sites we sampled. This method brought to our attention the existence of new sites, thereby giving us specific keywords to use in the Second Life search engine, or provided direct links to the sites themselves. This suggests that the Second Life search engine is more effective when specific keywords are entered or when the user is already familiar with the name of the organization hosting the site. It also suggests that there are barriers to finding health-related information on Second Life using typical Internet strategies, such as search engines. More likely, users are drawn to these sites through Second Life advertising, Second Life special events, referrals from other sites, or networking with other users who recommend visiting the site.

 We found a considerable number of Second Life sites (34) whose primary purpose is to disseminate health information. There are many features of Second Life that make it an ideal tool to do so. The interactivity, online accessibility, dynamic visual displays, and communication capabilities are key components in many of the health sites we surveyed. As mentioned previously, we tracked the popularity of the size of the sites on the day of December 18, 2008. We did not find a particular correlation between the size of the site and its popularity; in fact, some sites that are smaller geographically had a higher traffic rating than larger sites. It is interesting to note that although there are some government sponsored sites, they are not necessarily the most popular. Our sample indicates that sites in the Support category and Education & Awareness category are notably popular.

The following is a summary of our findings supported by specific examples from sites we found to be compelling and particularly innovative.

### Education & Awareness

Of the 68 relevant health sites we surveyed in Second Life, 34 focused primarily on education and awareness. Educational activities include real-world health communication tools such as prepared messages disseminated through interactive information kiosks, poster and bulletin boards, broadcast multi-media productions such as health videos, slideshows, and presentations, and links to Web pages. Other sites provide Web interfaces for interactive health information seeking, such as search engines, database portals, and townhall style meetings. There are other interactive features such as games, simulations with user-participation, virtual labs and classrooms, and vicarious avatar experiences.

**Table 2:** Selection of health education and awareness sites (top 15 sites — ranked by traffic and, in addition, the University of Plymouth (Sexual Health Sim) which is discussed in article). See Appendix 1 for the complete results for this category

Name	Identified	First Accessed	Features	Traffic	Region Size (m^2^)	Region Name
CF University (Cystic Fibrosis University)	CLR	Oct 30 2008	Several social areas and meeting places, library, memorial, Art Gallery, Medical Center, theatre.	2205	57264	Boomer Island
Contact a Family – For Families with Disabled Children	CLR	Nov 26 2008	Information for parents about raising children with disabilities. Site visitors can send questions to the site's parent advisor.	608	784	Aloft Nonprofit Commons
Venus Ventures (Hottie Hospital)^a^	CLR^b^ & SLSRCH	Sept 2008	Mature site. Information about reproductive systems mostly presented in a pornographic way.	564	2784	Waved
Karuna	CLR^b^ & SB	Dec 1 2008	Grand opening Dec 1 2008 to commemorate the 20th anniversary of World Aids Day. AIDS education and awareness, classroom, auditorium, links to other sites, social settings.	502	57920	Karuna
Healthinfo Island	SLSRCH	Sept 2008	Features a medical library (aka Second Life Medical Library) and consumer library, AIDS/HIV center, games, interactive displays, videos and more. Site run by RL librarians.	232	28528	Healthinfo Island
CDC Island	CLR^b^ & SLSRCH	Sept 2008	Information about various public health issues, links to external websites, virtual microbiology labs, conference rooms, information about the CDC.	224	63296	CDC Island
Virtual Hallucinations	CLR^b^ & SLSRCH	Sept 2008	Simulation of common hallucinations experienced by people with schizophrenia; focus on keywords, hearing voices, self-deprecating feelings, and more.	211	2560	Sedig
Ohio University Second Life Campus (Nutrition Game)^a^	CLR	Nov 4 2008	The Nutrition Game is one component of the Island (Featured Game). Interactive game teaches healthy food choices and nutrition.	179	53664	Ohio University
Autistic Liberation Front	CLR	Oct 30 2008	Hosted by an autism self-advocacy group. Meeting areas, memorial for autistic children who have been murdered, library/museum, interactive displays, store with SL items.	177	9360	Porcupine
Preferred Family Healthcare Island	CLR	Nov 4 2008	Real-life prevention and treatment provider for mental health issues and substance use. Online staff members, education, presentation areas, fitness center, conference rooms, game area.	175	62416	Preferred Family HC
Genome Island	CLR	Sept 2008	Scientific exploration of genetics. Scavenger hunt, many interactive features, free t-shirt of “your favourite chromosome” for avatars.	164	61264	Genome
Occupational Therapy Center at Thomas Jefferson University	SB	Nov 17 2008	Information about the role of occupational therapists. Interactive house display explains accessibility for physical and cognitive disabilities.	161	1120	Eduisland II
Tox Town at Virtual NLM	SB	Nov 17 2008	Includes a town, city, farm, port, and US-Mexico border, to help users identify toxic substances in their environment.	109	65536	Virtual NLM
Alliance for Consumer Education (ACE)	CLR^b^ & SLSRCH	Nov 20 2008	Information center for disease prevention and inhalant abuse prevention. Interactive “Stop Germs” House.	101	65536	ConsumerEd Island
Coordinated School Health for Teachers	SLSRCH	Nov 24 2008	Information for teachers on implementing health programs into education. (Also for general public.)	49	8192	Eduisland 5
University of Plymouth (Sexual Health Sim)	CLR^b^ & SLSRCH	Sept 2008	Information specific to sexual health; pictures of STD symptoms, interactive AIDS map, virtual condom, chatbot, quizzes, links, 3-D Tour of the Testes.	3	1536	Education UK

^a^Indicates that traffic and area size are bundled by the host, and include unrelated activities.

^b^Represents the first point of identification for the site.

### Healthinfo Island

Healthinfo Island is funded by a $40,000 grant [7] from the Greater Midwest Region of the National Networks/National Library of Medicine. The site is run by real-life health librarians and medical experts [7] and aims to provide users with education and awareness of and access to health information. The island features a Medical Library, a Consumer Health Library, PubMed search capability, the iVinnie Accessibility Center, and various other displays and meeting spots. There is a broad range of health information conveyed by kiosks in the Information Outreach Lab, from posters that redirect users to an HIV awareness website to ToxTown, an interactive area that explains the health risks from environmental agents.

According to van den Breckel (2007), Healthinfo Island aims to become the “central point in (Second Life) for health and medical information” [14] (page 1) by acting in cooperation with other health and medical agencies to “reach out to all Support Groups in Second Life” [14] (page 1). Healthinfo Island’s Pathway of Support serves as a clearinghouse for support group activity, providing users with information about various health-related support groups in the virtual world. Healthinfo Island is also committed to encouraging the development of support groups in Second Life, offering free land for six months to select non-profit health groups and organizations [14].

### University of Plymouth Sexual Health Sim

The Sexual Health Sim, run by the University of Plymouth (UK) contains public health information about sexually transmitted diseases. The site was made possible by a land grant from Education UK in July 2007 [15] and contains several interactive features, such as photographs of symptoms of various sexually transmitted diseases and a 3-D tour of the testes. Users can read about condoms and safe sex practices, and receive a virtual condom for their avatars to use. Avatars can also simulate the experience of illness by literally donning a “skin” (similar to clothing but acts like a second skin) that, in this case, visually displays the lesions of AIDS-related Kaposi Sarcoma on the avatar. Furthermore, the Sim provides information about both active sexuality and abstinence groups. The Sexual Health Sim employs unique features of Second Life in order to communicate health information to users.

### CDC Island

CDC Island is a 3-D virtual representation of the US Centers for Disease Control and Prevention. The island, which neighbors Healthinfo Island, contains many displays that link users to different websites and, at times, allows them to participate in discussion and focus groups. The buildings are interactive and contain meeting rooms, reception areas, and even microbiology labs where users can interact with microscopes to examine different bacteria and diseases. John Anderton, one of the creators of CDC Island, stated that he wanted the island to be a place for information, education, and dialogue [16]. The site includes several outreach activities, including CDC robots that ask for comments and site suggestions, a bracelet for avatars that automatically informs users of health awareness initiatives, live RSS feeds of health stories, and occasionally a live CDC representative is available in-world.

The National Institutes of Health (NIH) Second Life White Paper noted the benefit of anonymity for users in seeking health information and the opportunity to speak directly with a CDC representative [17]. Anderton notes that these representatives direct people to the information they need and are not “a surrogate for doctor-patient information” [18].

### Women’s Health Center at the Ann Myers Medical Center

The Ann Myers Medical Center, founded by Dr. Ann Buchanan in honor of her mother, Ann Myers, is run entirely by real-life nurses and physicians who donate their time. Much of the site is off limits to non-members. For its members, the site offers education through classrooms, resources, and simulations and is noted as being the first Second Life community to have adopted medical simulations in 2007 [19].

**Figure 1:**
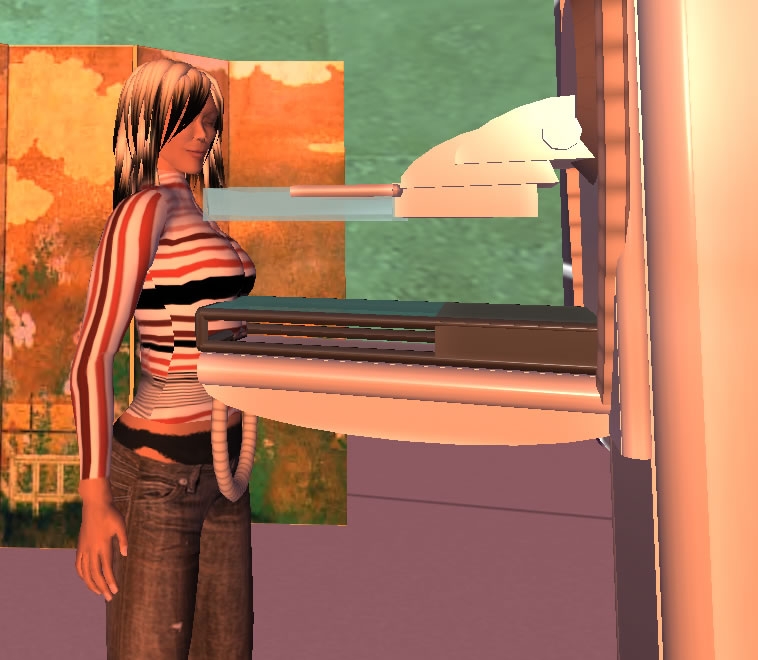
Our avatar Ellebee Helendale receiving a mammogram at the Ann Myers Medical Center

A few areas are open to all visitors, including the Women’s Health Center (WHC), where we received note cards explaining the importance of self-breast examinations. A room in the WHC shows female users how to perform their own breast exams. Another area shows what a mammogram machine looks like. As our avatar received a virtual mammogram (Figure 1), we were urged by our tour guide (a member of the AMMC) to take these lessons from SL (Second Life) to RL (Real life).

### Virtual Hallucinations

Virtual Hallucinations, a project originally launched by Peter Yellowlees and collaborators at the School of Medicine at the University of California at Davis, aims to educate people about the perceptual abnormalities experienced by schizophrenics by simulating common hallucination experiences. Auditory and visual hallucinations are simulated as the avatar enters the space and begins interacting with objects in the room. As our avatar entered the room, we were presented with a note card warning us not to proceed with the simulation if there was any history of mental illness in our family, indicating that the site managers are concerned about the real-life effects this site could have on users.

There are several interactive components within the simulation that aim to educate the user about this illness and the challenges faced by people with schizophrenia. In the pilot project, Yellowlees (2006) and his team surveyed 579 users who toured the site. Respondents were slightly more positive about the effects of the auditory hallucinations but indicated that the site improved their understanding of both auditory and visual hallucinations [20], and 82% of respondents said they would recommend the site to others [20]. Yellowlees thus shows that the virtual simulation was a successful education tool in promoting a deeper understanding of the experiences of those with mental illness.

The majority of health sites in Second Life offer some level of education and awareness. Our survey of health sites shows that Second Life offers unique and interactive ways to facilitate health education. The sites we found the most compelling are ones that took full advantage of experiential features.

### Support

Of the 68 relevant health sites in Second Life, 14 focused primarily on offering illness-specific patient or peer support. Many sites that aim to educate and promote awareness about specific illnesses also offer some level of individual and group support. Groups in Second Life offer support for everything from weight loss to living with AIDS. While these groups may not own virtual land or have their own spaces, they often will meet at other sites for discussions and will even host virtual events. Other sites offer personal consultations with health care providers and therapy sessions. Gorini stated that “3-D virtual worlds may convey greater feelings of presence, facilitate the clinical communication process, positively influence group processes and cohesiveness in group-based therapies, and foster higher levels of interpersonal trust between therapists and patients” [21] (page 1549). The interactivity and anonymity of Second Life make it an interesting platform for medical consultation, therapy, and peer support. Individual consultations and support groups are appropriate for Second Life largely due to the anonymity the platform encourages and by the many communication tools it provides. Some sites offer one-on-one appointments with doctors, nurses, medical librarians, therapists, and other health care professionals. Others provide virtual meeting places for groups to assemble and discuss the support group topic, moderated discussion groups, themed support group meetings, and group membership.

**Table 3:** Support sites (ranked by traffic)

Name	Identified	First Accessed	Features	Traffic	Region Size (m^2^)	Region Name
Transgender Resource Center – Peer Support & Social Activism	SB^a^& SLSRCH	Nov 17 2008	Offers support for transgendered individuals, regular meetings, peer support groups, online forums.	2313	1296	Aloft Nonprofit Commons
Wellness Island (Counseling Center – Homes and Offices)	CLR	Nov 5 2008	Library and support/education materials on mental health, relationships, etc. Counselling services available for a fee. Workshops also available. Community Outreach area provides links and direct teleports to other health sites in SL.	327	6528	Wellness Island
Live2Give	CLR	Nov 4 2008	Designed for people with severe physical limitations, to provide education, support, and a barrier-free environment.	192	65536	Live2Give
GimpGirl Community	CLR	Nov 18 2008	Meeting place for women with disabilities. Weekly support groups, presentations, and social gatherings.	66	15104	3DE
The Center for Positive Mental Health	CLR	Sept 2008	Provides support for various mental health issues. Managed by a psychologist. Book reviews and link to fee-based psychology appointments within SL. Meeting space for discussions.	43	5632	Kkotsam
Breast Cancer Network of Strength	CLR	Oct 30 2008	Site offers links and contact information for support groups, and meeting areas for people to discuss health issues.	29	4096	Association Works
NewWays – Counselling & Support	SLSRCH	Nov 17 2008	Offers free counselling and support to SL users by certified psychotherapists. Donations accepted. Confidentiality ensured, appointments available every Tuesday from 10:30am - 1:30pm PDT.	26	560	Hauwai
Meeting Circle	CLR^a^& SLSRCH	Nov 24 2008	Peer support groups with diverse health related talks; facilitated meetings several times per week. Notecards with some information about depression and medication, and suicide prevention.	24	1584	Support for Healing
12 Step Recovery Meeting Hall	SB^a^& SLSRCH	Nov 17 2008	Designed for any user going through a 12 step program (any addiction).	21	512	Idunn
Light Bearer Grief Center	CLR	Nov 4 2008	Room setting with links to support groups, grief resources, grief organizations, poems, music, other sites.	2	64	Imagination Island
Aspies for Freedom resource center	SB^a^& SLSRCH	Nov 17 2008	A meeting place for those diagnosed with Asperger’s syndrome. Note card links user to the website which holds forums, chatrooms, wikis and more.	2	960	Coders Cove
Autism Parent’s Connection–SOS	SLSRCH	Nov 27 2008	Site for parents of children with autism. Weekly meetings on Saturdays.	0	384	Amiaguas Avalon
The Counselling Center Annex Office	SB	Nov 17 2008	Information about individual health counselling. Users can make an appointment with a counsellor through the site. Description on “About” tab says it is the mainland satellite office for Wellness Island.	0	512	Boncarus
The Heron Sanctuary	CLR	N/A^b^	Meeting place for people with disabilities. Restricted access.	N/A	N/A	N/A

^a^Represents the first point of identification for the site.

^b^N/A indicates that the site was not available at time of sampling, or access was restricted (members only).

### Sexual Health Sim

The Sexual Health Sim offers links to outside support groups for people who have sexually transmitted diseases, AIDS, and HIV or questions about sexual health, as well as links to a Christian abstinence group and others. The site also holds its own discussion groups periodically, and one recent discussion group about disability and sexuality sparked quite a lot of interest in the Second Life community and was attended by more than 40 avatars [22].

### Transgender Resource Center — Peer Support & Social Activism

The Transgender Resource Center is a site that primarily offers support for transgendered individuals. The site is a room with chairs, a screen showing trailers for movies related to the subject (for example, “Transamerica”), and health information resources. The Center also holds regular meetings, peer support groups, and online forums for discussion. The popularity of the site should be noted. On almost every one of the authors’ visits to the site, they were greeted by other avatars using the space. On one visit, we met a group of avatars who were meeting socially in the site and were discussing transgendered issues outside of a discussion group. This site also had one of the highest traffic ratings of the sites we visited. The high traffic rating, coupled with the noted interactions in the space, suggest a popular and successful peer support group.

### Training

Of the 68 relevant health sites we discovered in Second Life, 11 focused primarily on training. The use of Second Life to provide specialized staff training is growing rapidly. IBM, Dell,and others are using the platform to their train staff, and several health care organizations have initiated training programs in Second Life. Several universities are training medical students in Second Life [23]. Stott (2007) noted that some universities have found that having a Second Life presence can affect the brand of the school and attract future students [23].

Typically, virtual training simulations provide users with an interactive and safe way to engage in a situation. Skiba (2007) quoted the Second Life Education Wiki in describing the benefits of using the platform for training and education: “Second Life provides an opportunity to use simulation in a safe environment to enhance experiential learning, allowing individuals to practice skills, try new ideas, and learn from their mistakes” [8] (page 156). Hansen (2008) argued that there are many gaps, unanswered questions, and potential issues with providing health care education in Second Life because very little empirical research has been conducted to suggest its efficacy [6]. However, the author felt that medical education in Second Life is something that should be pursued, and that research should be conducted to evaluate the value of the strategy and the effect of the experience of the users [6]. Training sites differ in their delivery of education, but most offer some level of simulation in which the users can participate. Many offer classes, classrooms, discussions, assignments, lectures, slideshows, videos, quizzes, and tests. Some users can even qualify for real-life credit for completing training scenarios in certain sites.

**Table 4:** Training sites (ranked by traffic)

Name	Identified	First Accessed	Features	Traffic	Region Size (m^2^)	Region Name
Ann Myers Medical Center	CLR^a^& SLSRCH	Sept 2008	Primarily used to train medical students. Public users can watch presentations, learn about health issues, and tour some of the virtual facilities with a member. Private areas include training resources for students, classrooms, presentations, conference rooms.	989	64192	Hospital
EMS Island	CLR	Dec 4 2008	Interactive quiz about medicine and health care, information about fractures, sprains, ailments, and diagnoses, links to external websites.	166	6656	Immaculate
Medical Examiner’s Office – Forensic Pathology	CLR	Nov 18 2008	Information about pathology, graphic images from dissections and autopsies. Area for virtual autopsy sim (not functioning at time of visit).	164	4096	San Miguel
University of Wisconsin-Milwaukee, Health Science	SLSRCH	Nov 24 2008	Classroom with instructions for a medical setting Scenario for avatars. Slideshow on ethics in health care.	124	8192	Arts and Letters
RL Education – Heart Murmur Sim	CLR	Nov 4 2008	“Cardiac Auscultation Training Concept”. Interactive activities with virtual patients.	101	1280	Waterhead
Evergreen Island	CLR	Dec 4 2008	Hosted by Washington State Community and Technical Colleges.Training area for nurses. MRI machine with explanation of it’s function. NHS signs. Patient rooms complete with bathrooms, classrooms with bed for avatar CPR, nurses station. Poster with a list of outcomes for SL class.	90	62400	Evergreen Island
CSCE – Healthcare Projects	CLR^a^& SLSRCH	Dec 11 2008	Designed as a hospital with Pharmacy, Patient Care area, Diagnostics, etc. Most interactive features not functioning at time of visit, but apparent that it is set up for training purposes.	88	24576	University of Arkansas
Imperial College London (Virtual Hospital)	CLR	Nov 5 2008	Virtual Respiratory Ward offers activities and simulated patient experiences. Students registered can receive course credit.	65	50784	Imperial College London
Medical Visualisation Network	CLR	Nov 18 2008	Aim is to produce new and innovative teaching solutions. Wall with pictures and bios of the Board Members of the MVN. Poster about virtual reality and anatomy training.	8	6832	Vue
SL Institute for Clinician Education (SLICE)	CLR	Nov 18 2008	University of Illinois virtual clinic for training medical students, physicians, and standardized patients.	0	512	Aido Wedo
Play2Train	CLR	Sept 2008	Emergency preparedness training simulation. Access restricted to members (invitation only). Designed in part to teach users how to manage patients and dispense drugs in emergency situations.	N/A^b^	N/A	N/A

^a^Represents the first point of identification for the site.

^b^N/A indicates that the site was not available at time of sampling, or access was restricted (members only).

### Imperial College London (Virtual Hospital)

Upon entry to the site we received an automated message saying our avatar had to register to be granted permission to enter the virtual hospital and treat patients. The Virtual Respiratory Ward offers activities and simulated patient experiences for which registered students can receive credit. Guests who have registered can participate but must pay for each diagnostic test they order for their virtual patient. The diagnostic simulation includes patient interviews, ordering diagnostic tests, arriving at a diagnosis, and providing treatment. This simulated environment allows students to go through the motions of visiting with a patient and the sequence of events that follows their treatment.

### Play2Train

Play2Train is a simulation sponsored by the United States Department of Health and Human Services [24] and supported by the Idaho Bioterrorism Awareness and Preparedness Program. The simulation trains users for emergency preparedness and features a virtual town and hospital where the training sessions take place [24]. Unlike other simulations, Play2Train forces participants to communicate with each other during sessions in a realistic fashion. The audio features of Second Life enhance the realism of the communication, as voice volume is dependent on the users’ proximity to one another [24]. The people behind Play2Train plan to compare the results of this training method to real-world simulations [24].

### Medical Examiner's Office — Forensic Path

The Medical Examiner’s Office provides information about pathology and how autopsies are performed. Graphic images from dissections and autopsies are the backdrop for an autopsy simulation in which the user’s avatar is the coroner. Although it was not fully functional at the time of our visit, our avatar was given tools to perform an autopsy on a virtual corpse. The simulation opportunities in Second Life are virtually limitless and can be built to provide training scenarios for many different purposes. Other features that allow lectures, virtual classrooms and assignments, videos, and slideshows also enable training within the platform.

### Marketing/Promotion of Health Services and Institutions

Of the 68 relevant health sites in Second Life, 6 focus primarily on marketing and the promotion of health services. The use of Second Life as a means of promoting business and emerging technologies is not exclusive to companies like IBM and Apple. Health care institutions are now using the platform as a way of promoting specific hospitals, services, health system reform, and even fundraising. It also provides a global showcase for best practices in medicine. The simulation capabilities in Second Life allow for organizations to provide users with first-hand virtual experiences of their initiatives and thereby garner public support. They can also recruit membership among Second Life users for real-life projects and promote upcoming fundraising initiatives.

**Table 5:** Marketing sites (ranked by traffic)

Name	Identified	First Accessed	Features	Traffic	Region Size (m^2^)	Region Name
American Cancer Society (Office and Lobby)^a^	CLR^b^ & SLSRCH	Oct 30 2008	Office area includes information on Relay for Life office, and several executive offices and conference rooms. Volunteer recruitment.	358	16416	American Cancer Society
Palomar West Hospital	SLSRCH	Sept 2008	PWH is a virtual replica of the new hospital being built in San Diego in 2011. It features a simulation of the future patient experience.	222	64112	Palomar West Hospital
Second Health (by Imperial College London)	CLR^b^& SLSRCH	Sept 2008	SH is affiliated with the NHS and has many different areas, including a Polyclinic Tour, auditorium, hospital, a training facility, and a private medical school.	49	64064	Second Health London
Diabetes UK	CLR	Nov 18 2008	Information about the organization (research charity), donation recruitment, “Diabetes Info Centre”, meeting areas, work stations, support phone number provided (RL).	18	5136	21CC
AICR (Association for International Cancer Research)	CLR	Oct 30 2008	Virtual auditorium, FAQ’s about different types of cancer, fundraising activities for cancer research (fashion shows, SL items)	3	22192	AICR
Faster Cures	CLR	Nov 26 2008	Information about the organization, curing diseases, innovations in treatment, and clinical trials.	3	640	Aloft Nonprofit Commons

^a^Region contains multiple health-related areas specific to the region; only one area was sampled and recorded.

^b^Represents the first point of identification for the site.

### Second Health (UK)

Second Health showcases recent efforts to implement the polyclinic model, a single point of access for both insured hospital and clinic medical services by simulating a London polyclinic. The region is set up like a town with several different areas to which users can teleport, such as a training area, a hospital, a polyclinic, and the Second Health Auditorium which hosted the first meeting for the international Virtual Association of Surgeons (iVAS) in April 2008. iVAS was attended by forty-seven avatars from five different countries [25]. Second Health has an extensive website which states that “The future of healthcare communication” [26]. Another part of their current outreach includes videos on YouTube that show various scenarios of the Second Life simulation, still allowing those who are not Second Life members to learn about Second Health.

### Palomar West Hospital

Palomar West Hospital (PWH) is a virtual simulation of a hospital which will be opened by Palomar Pomerado Health (PPH) in 2011 [27]. The Cisco-powered site, which is based on the blueprints for the future physical building [28] also showcases Cisco technologies that will be used within the hospital [29]. From the moment our avatar stepped into PWH, an automated yet interactive simulation began. When we entered, we were greeted by a woman on a large screen near the reception desk. She advised us that we would be wearing a hospital ID bracelet, equipped with an electronic identification tag, which assigned us to a health scenario: our avatar was informed that she required gall bladder surgery and that she must proceed to the elevator which will take her directly to her patient room. From this point on, our avatar was led through an extensive simulation and explanation of the design and experience of the patient room, diagnostic testing, robotic surgery procedures, and recovery. Orlando Portale, the Chief Innovation Officer of PPH, stated that the primary goal of creating PWH “was to allow our constituents to experience, rather than just to see the entire project” [30].

### American Cancer Society (ACS)

The ACS site in Second Life features entertainment areas, donations from visitors, a multi-level building with office space and meeting rooms, and information about the ACS and Relay for Life of Second Life. Relay For Life of Second Life, launched by the ACS in 2005, is a virtual walk-a-thon to raise money for the American Cancer Society. The event has grown substantially since its inception in 2005, when it raised nearly US $5,000. In 2007 it raised nearly US $120,000 [31], and the 2008 event, which was held on July 19, raised over US $200,000 [32]. Attendance for the event has also grown astronomically. In 2005, the event was attended by a few hundred avatars [31], while the 2008 event was attended by 2300 [32]. The office areas in the ACS site provide information about volunteering for Relay for Life of Second Life, complete with a bulletin board with job opportunities.

Second Life offers many features that can enhance an organization’s marketing initiatives. Traditional advertising is replicated in Second Life with billboard ads and product placement. Real-life current and future initiatives can also be replicated in the platform as simulations and user experiences. The variety of communication tools and interactivity provide organizations with new and innovative ways to reach out and gain buy-in from their clientele.

### Research

In our survey of health sites, we found few that focused primarily on conducting health research within Second Life (3 of 68 relevant health sites). Second Life offers the potential for health research to be conducted directly and indirectly within the platform. Bainbridge (2007) discusses the value of conducting research in virtual worlds, stating that they can create laboratories, experiments, and settings that simulate the real-world experience at a very low cost [33]. Furthermore, researchers could have access to a large population of subjects given the growing demographics of Second Life [33].

HHP at UH (Health & Human Performance at the University of Houston) in Second Life focuses on promoting healthy lifestyles. The site includes a large auditorium with video screen, presentations, and many buildings, including the Exercise Science building and the Texas Obesity Research building, it and offers visitors payment in Linden dollars to participate in surveys, studies, and activities. One of these studies is a 28-day health challenge for which avatars can enrol to participate.

Many sites give users note cards that contain voluntary surveys, asking questions about their experience, while others offer the user note cards to recruit participants for other studies. For example, the SL-Labs Psychology at University of Derby site offers users the chance to enrol in psychological studies.

Despite the lack of direct health research being conducted via avatar studies in Second Life, there are many other indirect ways that health care organizations are conducting research within the application. Second Life is also a viable resource for collecting passive research data and surveillance on various health topics, including what health issues users are researching, the geographical location of those searches, discussion topics, and health concerns. The CDC has been conducting in-world focus groups with avatars to collect data about the design and content of the virtual space [34]. Land owners can also keep track of site “traffic” (ie, the number of minutes avatars spend on the site) [12].

Second Life can also be used to survey the effectiveness of the information being displayed within the platform by tracking site referrals, visits to linked websites, and repeat visits from avatars. Van den Breckel (2007) states that one of the grant purposes of Healthinfo Island is to research the benefits and efficacy of disseminating health information within Second Life, stating, “All resources, areas and informational displays are being ‘monitored’ to evaluate effectiveness based on gathered statistics. Information on navigation, length of stay, items ‘touched’ will be used as input for this research” [14] (page 4).

**Table 6:** Health research sites (ranked by traffic)

Name	Identified	First Accessed	Features	Traffic	Region Size (m^2^)	Region Name
Stanford University Libraries^a^	SLSRCH	Nov 27 2008	Virtual Stanford Psychology Department where users can register to participate in experiments. Not functioning at time of visit.	2360	65536	Stanford University Libraries
Biomedicine Research Organization	SLSRCH	Nov 27 2008	Lecture area with slides on Chlamydia, research labs (purpose unknown), classrooms, board rooms, interactive display, virtual hospital. Links to information about the organization.	38	65536	Biomedicine Research Labs
HHP at UH (Health & Human Performance at the University of Houston)	CLR	Nov 5 2008	Offers visitors payment (in $Linden) to participate in surveys and activities, including a 28 day health challenge.	23	32224	HHP at UH

^a^Indicates that traffic and area size are bundled by the host, and include unrelated activities.

## Discussion

Our survey of health sites on Second Life indicates that virtual worlds have significant potential to improve health communication and patient experiences in the real world. Second Life is being used to educate users about important public health issues, train health care providers, market and promote health services, allow individuals to seek out individual or group support for diverse health issues, and, finally, to facilitate research. The steady rise in Internet use for seeking health information has converged with increased popularity of a range of Web 2.0 applications. These applications are increasingly returned when users perform keyword searches on health issues of interest. For example, a typical Google search of keywords from popular health topics will direct users not only to traditional websites, but also to YouTube videos, health blogs, and even to virtual worlds like Second Life. The Gartner Group consultancy claims that by 2011, 80% of active Internet users will use virtual realities [35]. In 2008, the McKinsey Group consultancy expressed the validity of Second Life and warned: “Ignore Second Life at your peril”[36].

Virtual worlds, like Second Life, offer unique didactic experiences to users seeking health information, skill building and health care training, group support, and, finally, individual consultation. Second Life venues for training can remove the travel and overhead costs that traditional health care training requires, and 3-D simulations can increase the utility of online training in areas where one-on-one inter-personal communication is an issue, as in the classic physical exam. While training programs on Second Life have a demonstrable utility, it is unclear how the communication of health information or virtual health experiences may impact individual health behavior. Research examining the impact of patient expectations and anxiety over health procedures suggests that this “priming” of patients with a Second Life experience may improve clinical outcomes by giving patients a better understanding of the health care system and its procedures before they come to the hospital or the clinic [3]. Having a virtual experience may give patients an increased sense of control over health experiences and should improve both knowledge and confidence, since the patient can navigate the health care system from the comfort of their own home. Patients can literally practice being patients or making healthy choices: they can formulate and ask questions in a simulated health experience and receive reinforcement from a variety of virtual experiences.

How experiences from Second Life might translate into real life is unclear and requires further research. Would having a trim, fit avatar have any impact on a person’s real self-image? Might it motivate people to engage in healthier behaviors? Second Life offers researchers unique opportunities to monitor user behavior and to study the impact of health communication, interventions, and training. Simulating a typical health experience scenario can make the logic of medical advice more comprehensible and concrete, for example by personalizing the long-term risks of certain health behaviors like smoking. Users can have their avatar experience the outcome of certain risk behaviors as a variety of illnesses, and their trust and responsiveness to public health recommendations may be bolstered by Second Life experiences. Thus, Second Life experiences have the potential to improve user trust in, and compliance to, public health messaging.

### The Real-life Implications of Second Life

Studies show that Second Life has real-life behavior implications. One study indicates that the behavior of users even correlates to the physical appearance of their avatars. Researchers at Stanford University’s Virtual Human Interaction Lab coined the term “Proteus Effect” to describe this phenomenon, as they found that the height of the avatar affected the users’ assertiveness and behavior within the virtual setting [3]. The appearance of the avatar alone can thus indicate some of the behaviors of the user.

National Institutes of Health (NIH) states that the Second Life platform enables public outreach initiatives and that it can be used by global health organizations as a new usability model for collaboration and to explore new ways to communicate health information [17]. Huang et al (2008) suggest that professional collaboration within Second Life may lead to real-life collaboration and exploration [37]. Many outreach initiatives for health care are being explored by various organizations, companies, and individuals in an effort to impact real-life behaviors.

Simulations that teach users about a specific topic can leave a lasting impact that transfers to the real world. In the Virtual Hallucinations pilot project, Yellowlees found that users reported a greater understanding of hallucinations and schizophrenia as a result of the simulation [20]. Web 2.0 applications such as Second Life have been credited for reducing the stigma of Autism and Asperger’s, as they promote awareness and stimulate empathy [38]. These are examples of knowledge transfer that will most likely follow users from Second Life to real life as they encounter health topics like schizophrenia and autism in the real world, they will have an awareness and understanding that was previously lacking and respond to situations differently as a result.

While some sites provide awareness about external topics, others look to provide the user with information that can be applied to their own real lives. Hoch, a neurologist at Massachusetts General Hospital, developed a pilot project to determine the effects of virtual meditation and relaxation on reducing real-life stress [39]. Hoch found that the virtual group displayed the same type of interactions as the real-life groups [30]. Bignell, a psychology lecturer at the University of Derby, England, is using Second Life to study how the platform can improve real-life communication skills among users with autistic spectrum disorders [40]. Gustafson (2008) stated that Second Life could potentially improve the results of substance abuse treatments, in part because the exposure to health information in Second Life may influence users to seek real-world information, advice, and treatment [3]. There is even a case report of a woman combating alcoholism within the platform. “Shelly”, and approximately 100 other alcoholics, underwent a therapy program through Accelerated Recovery Centers in Atlanta that existed solely in Second Life, where they were taught real-life techniques for avoiding alcohol [41]. Gustafson suggested that therapy for substance abuse in virtual reality, particularly virtual role-playing exercises, could have real-life benefits because users are able to practice new behaviors in a safe, simulated environment [3].

Some studies suggest that Second Life is a powerful research tool because it allows researchers to predict real-life behavior in a simulated setting [3,42,43]. Slater reproduced Stanley Milgram’s controversial shock therapy experiment where participants displayed a strong obedience to authority despite their own sentiments. Slater’s virtual reproduction of the experiment was not conducted in Second Life, but in a similar virtual setting using an avatar as a subject. Although the participants inducing the shocks to the avatar knew the experiment was not connected to a real person, they were still uncomfortable shocking the avatar. Despite their discomfort with the experiment, the users continued to shock the avatar, just as Milgram’s subjects had continued to shock their subject. Slater stated that virtual realities can thus be used as predictors for real-life human behavior [43].

Some agencies are building their sites specifically in an effort to change real-world behaviors. The CDC hopes that visitors to their site will apply what they learned in real life. Erin Edgerton, the content lead for interactive media at the National Center for Health Marketing for the CDC stated, “Today, the focus is less on the CDC as an agency and much more on specific health-related issues and on engaging visitors in virtual behaviors that might influence real-world health decisions” [30].

The transfer of Second Life to real-life behaviors has implications for health care. The experiential qualities of Second Life can be leveraged to promote the transfer of behaviors in Second Life to real-life. Edgerton stated that “when people practice health behaviors in a virtual world, they are more apt to perform them in the real world” [44]. Disseminating health information in the virtual reality could thus offer more effective health communication, reach a substantial number of people at once, and, in turn, produce real-life health results at low cost and with high impact.

The realism and social interaction within Second Life make it a viable venue for developing and testing new technologies that have implications outside of the Second Life platform. As more users embrace virtual worlds and the technology continues to evolve, issues over the ethics of virtual world research, user privacy, avatar informed consent [33], and intellectual property will have to be addressed.

### Conclusions

Second Life offers various interactive and innovative ways of communicating health information to a growing population of users. We developed five categories to describe the range of health-related activities online. The most common category was those sites whose primary purpose was to disseminate health information, followed by sites for peer-support, training of health care professionals, sites marketing and promoting health institutions and products, and, finally, sites dedicated to both virtual and real-world health research. The ability for individual users, health care institutions, and companies to create their own content presents opportunities for greater access to information and access to real-world health resources.

The design attributes of successful Second Life health sites suggest that both anonymity and interactivity are paramount. Second Life offers users the ability to interact with and speak to real people in real time while preserving their anonymity. They can consult with experts and other individuals with shared experiences, either privately or publicly in a group setting. Even when engaged in public discourse, there is still an element of privacy that does not exist in real-world interactions. This makes Second Life a potentially powerful tool for enabling discourse about personal and private issues, particularly those concerning health.

The number of health sites within Second Life indicates a need for this type of interaction in health care. Health care and communications professionals can learn why people are attracted to these virtual settings to engage in health discourse and potentially apply these lessons to real-world health communication strategies. Users are encouraged to expand their understanding of illnesses and to practice health promotion and prevention strategies through their avatar’s experiences. To be able to ask questions and pursue health information and experiences in an interactive 3-D setting, with inter-personal but anonymous contact, is singular to virtual worlds and opens up a range of exciting new possibilities for both patients and health care professionals.

## Multimedia Appendix 1

Full list of all identified sites.

## Abbreviations

AMMC: Ann Myers Medical Cente
CDC: Center for Disease Control
CLR: comprehensive literature review
HHP: Health & Human Performance
NIH: National Institutes of Health
RL: real life
SB: snowball
SL: Second Life
SLSRCH: Second Life Search Engine
UH: University of Houston
WHC: Women’s Health Center
