# Supplementary material for: A Survey of Health-Related Activities on Second Life
Source: J Med Internet Res. 2009 May 22;11(2):e17. doi: 10.2196/jmir.1192 (PMC2762804; doi:10.2196/jmir.1192)
Supplement: Supplementary file 1 [file jmir_v11i2e17_app1.pdf]

Multi-Media Appendix 1: [Full list of all identified sites. Search methods used to identify each site are coded as follows SLSRCH = Second Life Search Engine, CLR = Comprehensive Literature Review, SB = Snowball. Bold abbreviations represent the first point of identification for the site. The date we first accessed each site is listed. Data for "Traffic" and "Region Size" were collected in a randomized order, on December 18, 2008. The "Classification" represents the authors' own categories of sites. Where multiple classifications apply, the primary classification is listed first, in bold. "Traffic" is a rough measure of the popularity of the site (the figure is based on a Second Life algorithm where the higher number is, the higher popularity); "Region size" is measured in m2. Exceptions are indicated as follows: *Region contains multiple health-related areas specific to the region; only one area was sampled and recorded **Indicates that traffic and area size are bundled by the host, and include unrelated activities. N/A indicates that the site was not available at time of sampling, or access was restricted (members only).]

Name	Identified	First Accessed	Features	Traffic	Region Size	Region Name	Classification
Stanford University Libraries**	SLSRCH	Nov 27 2008	Virtual Stanford Psychology Department where users can register to participate in experiments. Not functioning at time of visit.	2360	65536	Stanford University Libraries	**Research**, Education & Awareness, Marketing
Transgender Resource Center - Peer Support & Social Activism	SB & SLSRCH	Nov 17 2008	Offers support for transgendered individuals, regular meetings, peer support groups, online forums.	2313	1296	Aloft Nonprofit Commons	**Support**, Education & Awareness
CF University (Cystic Fibrosis University)	CLR	Oct 30 2008	Several social areas and meeting places, library, memorial, Art Gallery, Medical Center, theatre.	2205	57264	Boomer Island	Education & Awareness
Ann Myers Medical Center	CLR & SLSRCH	Sept 2008	Primarily used to train medical students. Public users can watch presentations, learn about health issues, and tour some of the virtual facilities with a member. Private areas include training resources for students, classrooms, presentations, conference rooms.	989	64192	Hospital	**Training**, Education & Awareness
Contact a Family - For Families with Disabled Children	CLR	Nov 26 2008	Information for parents about raising children with disabilities. Site visitors can send questions to the site's parent advisor.	608	784	Aloft Nonprofit Commons	Education & Awareness
Venus Ventures (Hottie Hospital)**	CLR & SLSRCH	Sept 2008	Mature site. Information about reproductive systems mostly presented in a pornographic way.	564	2784	Waved	Education & Awareness

Karuna	CLR & SB	Dec 1 2008	Grand opening Dec 1 2008 to commemorate the 20th anniversary of World Aids Day. AIDS education and awareness, classroom, auditorium, links to other sites, social settings.	502	57920	Karuna	Education & Awareness
American Cancer Society (Office PART A)*	CLR, SLSRCH	Oct 30 2008	Office includes information on Relay for Life office, and several executive offices and conference rooms. Volunteer recruitment.	342	11040	American Cancer Society	**Marketing**, Education & Awareness
Wellness Island (Counseling Center - Homes and Offices)	CLR	Nov 5 2008	Library and support/education materials on mental health, relationships, etc. Counselling services available for a fee. Workshops also available. Community Outreach area provides links and direct teleports to other health sites in SL.	327	6528	Wellness Island	**Support**, Education & Awareness
Healthinfo Island	SLSRCH	Sept 2008	Features a medical library (aka Second Life Medical Library) and consumer library, AIDS/HIV centre, games, interactive displays, videos and more. Site run by RL librarians.	232	28528	Health Infoisland	Education & Awareness
CDC Island	CLR & SLSRCH	Sept 2008	Information about various public health issues, links to external websites, virtual microbiology labs, conference rooms, information about the CDC.	224	63296	CDC Island	**Education & Awareness**, Marketing
Palomar West Hospital	SLSRCH	Sept 2008	PWH is a virtual replica of the new hospital being built in San Diego in 2011. It features a simulation of the future patient experience.	222	64112	Palomar West Hospital	Marketing
Virtual Hallucinations	CLR & SLSRCH	Sept 2008	Simulation of common hallucinations experienced by people with	211	2560	Sedig	Education & Awareness

			schizophrenia; focus on keywords, hearing voices, self depreciating feelings, and more.				
Live2Give	CLR	Nov 4 2008	Designed for people with severe physical limitations, to provide education, support, and a barrier-free environment.	192	65536	Live2Give	Support
Ohio University Second Life Campus (Nutrition Game)**	CLR	Nov 4 2008	The Nutrition Game is one component of the Island (Featured Game). Interactive game teaches healthy food choices and nutrition.	179	53664	Ohio University	Education & Awareness
Autistic Liberation Front	CLR	Oct 30 2008	Hosted by an autism self-advocacy group. Meeting areas, memorial for autistic children who have been murdered, library/museum, interactive displays, store with SL items.	177	9360	Porcupine	Education & Awareness, Support
Preferred Family Healthcare Island	CLR	Nov 4 2008	Real-life prevention and treatment provider for mental health issues and substance use. Online staff members, education, presentation areas, fitness center, conference rooms, game area.	175	62416	Preferred Family HC	**Education & Awareness**, Support
EMS Island	CLR	Dec 4 2008	Interactive quiz about medicine and health care, information about fractures, sprains, ailments and diangoses, links to external websites.	166	6656	Immaculate	Training
Genome Island	CLR	Sept 2008	Scientific exploration of genetics. Scavenger hunt, many interactive features, free tshirt of "your favourite chromosome" for avatars.	164	61264	Genome	Education & Awareness

Medical Examiner's Office -- Forensic Pathology	CLR	Nov 18 2008	Information about pathology, graphic images from dissections and autopsies. Area for virtual autopsy sim (not functioning at time of visit).	164	4096	San Miguel	Training
Occupational Therapy Center at Thomas Jefferson University	SB	Nov 17 2008	Information about the role of occupational therapists. Interactive house display explains accessibility for physical and cognitive disabilities.	161	1120	Eduisland II	Education & Awareness
University of Wisconsin-Milwaukee, Health Science	SLSRCH	Nov 24 2008	Classroom with instructions for a medical setting Scenario for avatars. Slideshow on ethics in health care.	124	8192	Arts and Letters	Training
Tox Town at Virtual NLM	SB	Nov 17 2008	Includes a town, city, farm, port, and US-Mexico border, to help users identify toxic substances in their environment.	109	65536	Virtual NLM	Education & Awareness
Alliance for Consumer Education (ACE)	CLR & SLSRCH	Nov 20 2008	Information centre for disease prevention and inhalant abuse prevention. Interactive "Stop Germs" House.	101	65536	ConsumerEd Island	**Education & Awareness**, Marketing
RL Education - Heart Murmur Sim	CLR	Nov 4 2008	"Cardiac Auscultation Training Concept". Interactive activities with virtual patients.	101	1280	Waterhead	Training
Evergreen Island	CLR	Dec 4 2008	Hosted by Washington State Community and Technical Colleges. Training area for nurses. MRI machine with explanation of it's function. NHS signs. Patient rooms complete with bathrooms, classrooms with bed for avatar CPR, nurses station. Poster with a list of outcomes for SL class.	90	62400	Evergreen Island	Training

CSCE - Healthcare Projects	CLR & SLSRCH	Dec 11 2008	Designed as a hospital with Pharmacy, Patient Care area, Diagnostics, etc. Most interactive features not functioning at time of visit, but apparent that it is set up for training purposes.	88	24576	University of Arkansas	Training
GimpGirl Community	CLR	Nov 18 2008	Meeting place for women with disabilities. Weekly support groups, presentations, and social gatherings.	66	15104	3DE	Support
Imperial College London (Virtual Hospital)	CLR	Nov 5 2008	Virtual Respiratory Ward offers activities and simulated patient experiences. Students registered can receive course credit.	65	50784	Imperial College London	Training, Marketing
Coordinated School Health for Teachers	SLSRCH	Nov 24 2008	Information for teachers on implementing health programs into education. (Also for general public.)	49	8192	Eduisland 5	Education & Awareness
Second Health (by Imperial College London)*	CLR & SLSRCH	Sept 2008	SH is affiliated with the NHS and has many different areas, including a Polyclinic Tour, auditorium, hospital, a training facility, and a private medical school.	49	64064	Second Health London	Marketing, Training
The Center for Positive Mental Health	CLR	Sept 2008	Provides support for various mental health issues. Managed by a psychologist. Book reviews and link to fee-based psychology appointments within SL. Meeting space for discussions.	43	5632	Kkotsam	Support
Biomedicine Research Organization	SLSRCH	Nov 27 2008	Lecture area with slides on Chlamydia, research labs (purpose unknown), classrooms, board rooms, interactive display, virtual hospital. Links to information about the organization.	38	65536	Biomedicine Research Labs	**Research**, Education & Awareness, Marketing

MS Island Nederland	CLR	Nov 4 2008	Based in Amsterdam. MS Information Centre, several social areas including a dance hall, meditation areas.	32	65456	MS Island Vumc	Education & Awareness
Autism Society of America	SB & SLSRCH	Nov 17 2008	Weekly meetings every Saturday at 5pm SLT. (All SL users welcome.) Various meeting places and information videos, Autism Information Library	30	32768	eRa	Education & Awareness
Breast Cancer Network of Strength	CLR	Oct 30 2008	Site offers links and contact information for support groups, and meeting areas for people to discuss health issues.	29	4096	Association Works	Support
NewWays - Counselling & Support	SLSRCH	Nov 17 2008	Offers free counselling and support to SL users by certified psychotherapists. Donations accepted. Confidentiality ensured, appointments available every Tuesday from 10.30am-1.30pm PDT.	26	560	Hauwai	Support
Meeting Circle	CLR & SLSRCH	Nov 24 2008	Peer support groups with diverse health related talks; facilitated meetings several times per week. Note-cards with some information about depression and medication, and suicide prevention.	24	1584	Support for Healing	Support
HHP at UH (Health & Human Performance at the University of Houston)	CLR	Nov 5 2008	Offers visitors payment (in $Linden) to participate in surveys and activities, including a 28 day health challenge.	23	32224	HPP at UH	**Research**, Education & Awareness, Marketing
Virtual Cancer Institute	SLSRCH	Nov 27 2008	Site hosted the Breast Cancer Virtual Convention in Nov 08 (attended by the CEO of Linden Lab, Mark Kingdon). Large lecture auditoriums with screens, boardrooms with screens, classroom	21	1936	ISN Visions	Education & Awareness

			(last class was called "Advanced Course on Breast Cancer").				
12 Step Recovery Meeting Hall	SB & SLSRCH	Nov 17 2008	Designed for any user going through a 12 step program (any addiction).	21	512	Idunn	Support
Diabetes UK	CLR	Nov 18 2008	Information about the organization (research charity), donation recruitment, "Diabetes Info Centre", meeting areas, work stations, support phone number provided (RL).	18	5136	21CC	**Marketing**, Education & Awareness
SL-Labs Psychology at University of Derby (1)	SLSRCH	Nov 17 2008	Presentation area with slideshow, group discussions encouraged, learning activities throughout the space. Offers open membership to various research groups.	17	8448	Games at DerbyUni	Education & Awareness, Research
Veterans Health Administration (VHA)	CLR	Nov 18 2008	Information for veterans regarding health care benefits. Links to websites with info about facilities, programs, charges, prescriptions, downloadable PDFs, video on suicide prevention. Meeting room with projector and screen.	14	1632	depo business hub	**Education & Awareness**, Support
Health Eduisland	SB	Nov 17 2008	PubMed displays with abstracts and links to articles, "Second Life and Public Health" movie, AIDS info area, display of current projects, poster with Design Goals.	12	51328	Health Eduisland	Education & Awareness
Medical Visualisation Network	CLR	Nov 18 2008	Aim is to produce new and innovative teaching solutions. Wall with pictures and bios of the Board Members of the MVN. Poster about virtual reality and anatomy training.	8	6832	Vue	Training
Drug Policy Alliance Network	CLR & SLSRCH	Nov 26 2008	Very small site with information and links to pdfs about different	5	320	Aloft Nonprofit Commons	**Education & Awareness**, Marketing

			drugs. (Group is against the War on Drugs)				
SL HIV Prevention and Education Center	CLR	Nov 5 2008	Site provides education and awareness about HIV and AIDS. Links to various websites, condoms for sale for avatars, support.	4	288	Madhupak	**Education & Awareness**, Support
University of Plymouth (Sexual Health Sim)	CLR & SLSRCH	Sept 2008	Information specific to sexual health; pictures of STD symptoms, interactive AIDS map, virtual condom, chatbot, quizzes, links, 3D Tour of the Testes.	3	1536	Education UK	Education & Awareness
AICR (Association for International Cancer Research)	CLR	Oct 30 2008	Virtual auditorium, FAQ's about different types of cancer, fundraising activities for cancer research (fashion shows, SL items)	3	22192	AICR	**Marketing**, Education & Awareness
Faster Cures	CLR	Nov 26 2008	Information about the organization, curing diseases, innovations in treatment, and clinical trials.	3	640	Aloft Nonprofit Commons	**Marketing**, Education & Awareness
MS Society	CLR	Nov 4 2008	MS Fly, ads for "Moving Forward Film Festival" (March 08), links to educational websites, donation box, videos.	2	1728	Illusion Factory West	**Education & Awareness**, Marketing
Cies Island - Survivors of TBI	CLR	Nov 5 2008	Information about Traumatic Brain Injury. Site has an MD and RT on staff. Free wheelchairs for avatars, and a multimedia message board.	2	4352	Cies Island	**Education & Awareness**, Support
Light Bearer Grief Center	CLR	Nov 4 2008	Room setting with links to support groups, grief resources, grief organizations, poems, music, other sites.	2	64	Imagination Island	Support
Aspies for Freedom resource center	SB & SLSRCH	Nov 17 2008	A meeting place for those diagnosed with Asperger's syndrome. Note card links user to the website which holds forums, chatrooms, wikis and more.	2	960	Coders Cove	**Support**, Education & Awareness

BCA Garden of Hope, Peace & Love	CLR	Oct 30 2008	Breast cancer awareness garden. Chapel with memorial, landmarks to other sites, information about breast cancer.	1	2048	EDEL	Education & Awareness
Iowa Wellness and Spinal Tuning Center	SLSRCH & CLR	Sept 2008	Ads for hiring a RL doctor, and information about various chiropractic issues. Weekly lectures in Vaccine911 Lecture Hall (anti-vaccine presentations).	1	688	Iowa	**Education & Awareness**, Marketing
MD Kiosk	CLR	Nov 4 2008	Several free classes with videos and diagrams on different health topics. Also offers some classes that require payment. Classes cost between L$137 - L$180.	0	28320	MD Kiosk	Education & Awareness
National Council for Community Behavioural Healthcare	CLR	Nov 26 2008	Interactive health quiz that offers free gifts for answering correctly; information on how to create a survival box (for people with suicidal tendencies); links to mental health websites.	0	1232	Aloft Nonprofit Commons	Education & Awareness
Solsbury Hill MS Resource Centre	CLR	Nov 5 2008	Information on worldwide Multiple Sclerosis organizations, meeting areas, massages for avatars. Free t-shirts for avatars and links to websites.	0	1024	Trompton	Education & Awareness
UIS/CSU/Basuah Welcome (University of Illinois Springfield, Chicago State University, Illinois Department of Public Health.	SLSRCH	Nov 27 2008	HIV/Aids Prevention Training area hosted by the University of Illinois, Chicago State University, and the Illinois Department of Public Health. Interactive quiz about AIDS/HIV myths, links to websites, info about medications, symptoms.	0	320	Univ Illinois Spfld	Education & Awareness
Autism Parent's Connection – SOS	SLSRCH	Nov 27 2008	Site for parents of children with autism. Weekly meetings on	0	384	Amiaguas Avalon	Support

			Saturdays.				
The Counselling Center Annex Office	SB	Nov 17 2008	Information about individual health counselling. Users can make an appointment with a counsellor through the site. Description on "About" Tab says it is the mainland satellite office for Wellness Island.	0	512	Boncarus	Support
SL Institute for Clinician Education (SLICE)	CLR	Nov 18 2008	University of Illinois virtual clinic for training medical students, physicians, and standardized patients.	0	512	Aido Wedo	Training
School of Health and Social Care - Bournemouth University	CLR	Nov 3 2008	Links and direct SLURLS to other health sims in SL.	N/A	N/A	Education UK 2	Education & Awareness
The Walk of Life Natural Health Education Center	SLSRCH	Nov 24 2008	Information about alternative health: e.g., raw diets, "healthy mind", supplements, links to websites with information and books for sale, recipes, presented as posters.	N/A	N/A	N/A	**Education & Awareness**, Marketing
The Heron Sanctuary	CLR	N/A	Meeting place for people with disabilities. Restricted access.	N/A	N/A	N/A	Support
Play2Train	CLR	Sept 2008	Emergency preparedness training simulation. Access restricted to members (invitation only). Designed in part to teach users how to manage patients and dispense drugs in emergency situations.	N/A	N/A	N/A	Training
